# Modulation of ERK5 Activity as a Therapeutic Anti-Cancer Strategy

**DOI:** 10.1021/acs.jmedchem.3c00072

**Published:** 2023-04-01

**Authors:** Duncan C. Miller, Suzannah J. Harnor, Mathew P. Martin, Richard A. Noble, Stephen R. Wedge, Celine Cano

**Affiliations:** † Cancer Research Horizons Therapeutic Innovation, Newcastle Drug Discovery Group, Newcastle University Centre for Cancer, School of Natural and Environmental Sciences, Newcastle University, Bedson Building, Newcastle upon Tyne NE1 7RU, United Kingdom; ‡ Cancer Research Horizons Therapeutic Innovation, Newcastle Drug Discovery Group, Translational and Clinical Research Institute, Paul O’Gorman Building, Medical School, Framlington Place, Newcastle upon Tyne NE2 4HH, United Kingdom

## Abstract

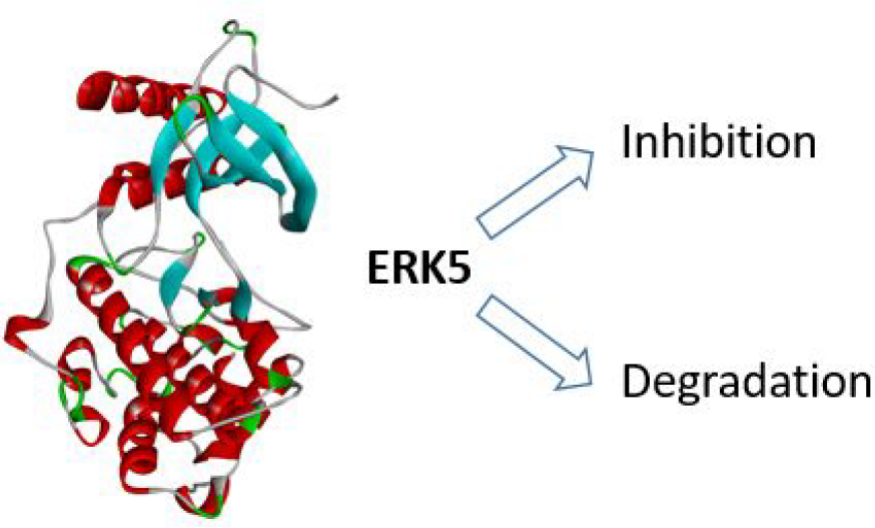

The extracellular signal-regulated kinase 5 (ERK5) signaling pathway is one of four conventional mitogen-activated protein (MAP) kinase pathways. Genetic perturbation of ERK5 has suggested that modulation of ERK5 activity may have therapeutic potential in cancer chemotherapy. This Miniperspective examines the evidence for ERK5 as a drug target in cancer, the structure of ERK5, and the evolution of structurally distinct chemotypes of ERK5 kinase domain inhibitors. The emerging complexities of ERK5 pharmacology are discussed, including the confounding phenomenon of paradoxical ERK5 activation by small-molecule ERK5 inhibitors. The impact of the recent development and biological evaluation of potent and selective bifunctional degraders of ERK5 and future opportunities in ERK modulation are also explored.

## Introduction

Extracellular signal-regulated kinase 5 (ERK5, BMK1, MAPK7) is one of four conventional mitogen-activated protein (MAP) kinases, which also include the ERK1/2, JNK1/2/3, and p38.^1^ MAP kinases are central to several pathologies including cancer. Vemurafenib, targeting B-Raf, and selumetinib and trametinib, targeting MEK1/2, have demonstrated the clinical utility of agents modulating MAP kinase pathways in cancer treatment. Since its discovery in 1995,^2,3^ the structure, function, and pharmacology of ERK5 have been extensively investigated.^1^ The structure of ERK5 differs significantly from those of the other conventional MAP kinases. Comprising 816 amino acid residues, ERK5 is twice the size of classical MAPKs^3^ and is consequently also known as Big Map Kinase-1 (BMK-1).^1^ The resulting multidomain structure leads to complex biological activity, which has been linked to roles in various disease states including cancer.^4,5^ This Miniperspective will discuss the structure and function of ERK5, the medicinal chemistry progress that has been made in inhibiting ERK5 kinase activity with small molecules, the pharmacological outcomes resulting from chemical-induced ERK5 degradation, and the collective implications for ERK5 modulation as an anticancer therapeutic strategy.

## Structure and Function of ERK5

The *N*-terminal kinase domain of ERK5 is highly conserved with other MAPKs, including ERK1/2, and contains the same TEY phosphorylation triad.^1^ Amino acids 1–77 at the *N*-terminus of ERK5 are involved in targeting the protein to the cytoplasm, with residues 78–406 comprising the kinase domain, which shares 66% sequence identity with ERK2. It is therefore important to understand the significant structural and mechanistic differences that lead to the marked differences in the pharmacology of ERK5 when compared to ERK1/2. ERK5 has a unique 410 amino acid *C*-terminal tail, which may have an autoinhibitory function, as truncation leads to increased kinase activity. The *C*-terminal tail also contains a nuclear localization sequence (NLS; residues 505–539) and a transcriptional activation domain (TAD; residues 664–789). These sequences confer the ability for ERK5 to directly regulate gene expression, which is unique in the MAP kinase protein family.^6^ Autophosphorylation of the TAD enables ERK5 to directly regulate gene transcription. The *C*-terminal tail of ERK5 also contains two proline-rich domains (PR1, residues, 429–464; PR2, residues 584–696), a nuclear export sequence (NES), and a myocyte enhancer factor 2 (MEF2)-interacting sequence.^7^

The ERK5 pathway may be activated by a wide range of extracellular stimuli, including multiple growth factors, inflammatory cytokines such as IL-6, and physical stimuli such as shear, osmotic, and hypoxic stresses.^1^ MEK5 (activated by the serine/threonine kinases MEKK2 and MEKK3) uniquely activates ERK5 by phosphorylation, first of T219 and subsequently of Y221 in a TEY amino acid triad.^8^ This activated form of ERK5 induces autophosphorylation of a number of its *C*-terminal residues and a conformational change that permits its nuclear translocation.^8−10^ It is then able to phosphorylate multiple downstream substrates including the transcription factors c-Myc, Mef2 A, C, and D, and c-Fos, along with other kinases such as ribosomal s6-kinase (RSK).^9^ ERK5 may also be activated for nuclear translocation through mechanisms independent of phosphorylation of the TEY triad, such as phosphorylation of *C*-terminal residues (T733, S720, and S754) by CDK1^11,12^ and phosphorylation of T733 by ERK1/2.^13^ Indeed, T733 has been described as a functional gatekeeper residue that controls *C*-terminal-mediated nuclear translocation.^14^ Sustained activation of upstream signaling may also result in dysregulation of ERK5 signaling.

**Figure 1:**
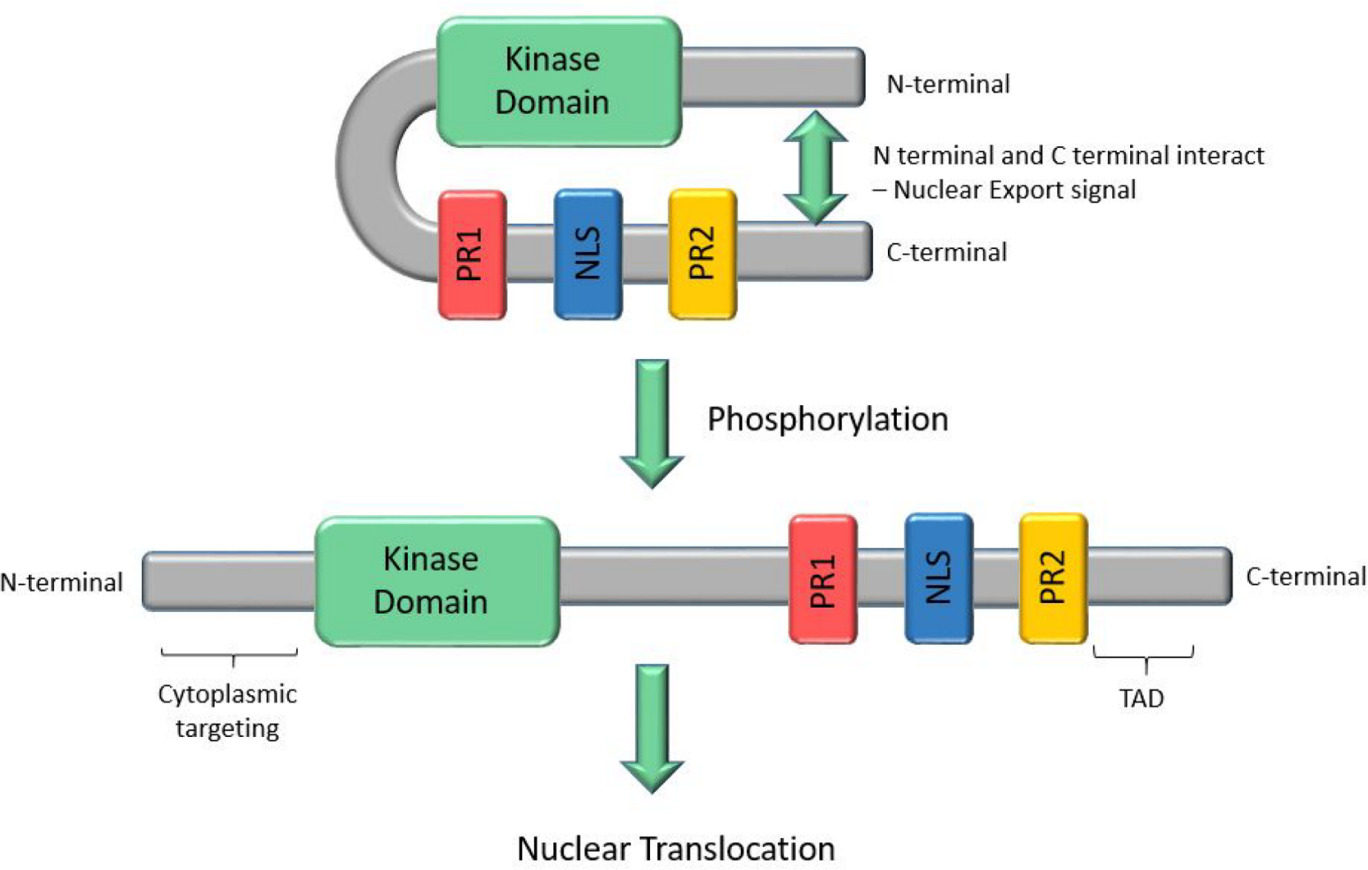
Schematic of the structure of ERK5 and the conformational changes that occur on phosphorylation, leading to nuclear translocation.

ERK5 and ERK1/2 both require translocation to the nucleus for downstream signaling, but the mechanism of control of translocation differs.^9^ It is proposed that the *C*- and *N*-termini of ERK5 interact in the inactive state, giving a folded conformation that is exported from the nucleus, an association that is disrupted by the aforementioned ERK5 *C*-terminal autophosphorylation (Figure 1). This contrasts with the translocation mechanism of the shorter ERK1/2 MAP kinases, which lack the extensive *C*-terminal domain of ERK5. MEK1/2 contains a nuclear export signal, and in its unphosphorylated form associates with ERK1/2, localizing it to the cytoplasm.^9,15,16^ On phosphorylation of ERK1/2, the ERK1/2-MEK1/2 complex dissociates, enabling ERK1/2 to translocate to the nucleus.

The mechanism of control of transcription by ERK1/2 also differs from that of ERK5.^9^ While ERK5 has a *C*-terminal TAD which is activated by autophosphorylation,^6^ ERK1/2, lacking such a domain, only acts by phosphorylation of other transcription factors.

ERK5 and ERK1/2 share several common phosphorylation substrates including Sap1a, c-Myc, RSK, c-Fos, and c-Jun. ERK5 also activates members of the MEF family of transcription factors which may induce apoptosis in specific settings, such as in neurotrophin-induced medulloblastoma cell death.^17^ MEF2D appears to be a specific substrate of ERK5, whereas MEF2A and MEF2C are activated by both ERK5 and p38 MAP kinases. Thus, MEF2D transcriptional activity is commonly used in reporter systems for examining ERK5 activity.^18^ An ERK5 construct containing only the kinase domain (AAs 1–400) was unable to activate the lklf (lung kruppel-like factor) reporter gene, whereas a construct devoid of kinase activity, comprising only the *C-*terminal residues (400–806), constitutively activated lklf transcription.^19^ This suggests that the kinase domain may have a regulatory function. ERK5 can also differentially activate transcriptional responses through phosphorylation when compared to other MAP kinases. For example, while ERK5 and ERK1/2 can both phosphorylate c-Fos,^9^ a transcription factor implicated in proliferation, differentiation, and apoptosis,^20^ only phosphorylation by ERK5 at multiple sites results in full c-Fos transcriptional activity. ERK5 also affects the transcription factors c-Myc, CREB, and Sap1a and indirectly affects AKT phosphorylation, all of which have been implicated in tumor development.^1^

## Genetic Perturbation of ERK5

A range of studies involving genetic perturbation of ERK5 with siRNA/shRNA-mediated gene silencing or deletion of the *MAPK7* gene (encoding ERK5) suggests that ERK5 signaling has a role in driving several distinct cellular phenotypes. Gene silencing of ERK5 has been shown to be antiproliferative in vitro or in vivo in a number of cancer cell types, including endometrial cancer,^21^ nonsmall cell lung cancer (NSCLC),^22^ small cell lung cancer (SCLC),^23^ melanoma,^24^ triple-negative breast cancer,^25^ and cholangiocarcinoma.^26^ In SCLC, a reduction in tumor cell proliferation upon ERK5 shRNA knockdown was not rescued by kinase-dead ERK5, in contrast to wild-type ERK5, suggesting that ERK5 kinase activity is important in mediating proliferation in this tumor type.^23^ A study in HeLa cells also linked an ERK5-dependent proliferative response to an upstream growth factor stimulus with the activation of ERK5 being induced by epidermal growth factor (EGF) treatment.^27^ Despite these observations, the proliferation of many tumor cells is not impacted by the genetic perturbation of ERK5,^28^ including cells where ERK5 is overexpressed or *MAPK7* is amplified.^29^ There are also reports of ERK5 perturbation with shRNA stimulating the growth of breast cancer xenografts.^30,31^ Clearly, while there is experimental evidence linking ERK5 to tumor cell proliferation in some cell models, it is not a universal phenomenon.

ERK5 gene knockout studies in mice have shown it to have an indispensable role in angiogenesis and heart development, with ERK5 loss resulting in cardiac defects and embryonic lethality.^32^ Conditional ERK5 gene knockout studies in adult mice have also shown it to be critical for the maintenance of normal vascular integrity, the induction of endothelial cell apoptosis, and vascular leakage, leading to death within a period of 2–3 weeks.^32^ The protective effect of ERK5 on endothelial cell homeostasis is thought to be dependent upon the laminar shear stress induced by a constant blood flow.^4^ Conditional ERK5 gene knockout also inhibits tumor angiogenesis, preventing the formation of functional blood vessels to a tumor site and consequently reducing the growth of tumor xenografts.^33^

ERK5 is also implicated as having a role in tumor cell invasion, with its activity in triple-negative breast cancer cell lines maintaining the expression of the transcription factor Slug, which represses genes involved in an epithelial-to-mesenchymal transition.^34^ Furthermore, ERK5 has been shown to induce the phosphorylation of FAK to promote cell motility but in an ERK5 kinase-independent manner.^31^ Consistent with these findings, ERK5 silencing has been shown to reduce the invasion of breast tumor cell lines in vitro and the metastasis of breast cancer xenografts in vivo.^34,31,35^

Other cancer cell phenotypes that are reportedly induced by genetic perturbation of ERK5 include reduced tumor cell lipid metabolism,^23^ an increase in autophagy,^36^ and the induction of cellular senescence.^37^ Experimental evidence also suggests that ERK5 inhibition has the potential to impact the inflammation and activation of the immune system by reducing the secretion of interleukin-6 (IL-6) from different cell types,^38,39^ regulating macrophage polarization to become less tumor supportive (M2-like),^40^ reducing CSF-1-stimulated macrophage proliferation, and increasing tumor cell chemokine expression to induce T-cell infiltration.^41,42^

Use of ERK5 siRNA also suggests ERK5 inhibition may have utility in combination regimens, either with DNA-damaging cancer chemotherapy or with radiation through the modulation of DNA repair,^43,44^ or other molecular targeted approaches, including acquired resistance to combined MAPK pathway inhibition.^16,45^

Collectively, inhibition of ERK5 using gene silencing and deletion approaches has implicated the protein as having a role in a diverse range of signaling responses relevant to cancer. These experiments provided the impetus for the discovery of orthosteric ERK5 kinase inhibitors with considerable effort being employed to develop selective tool compounds that were suitable for examination in biological systems. However, small-molecule inhibitors of ERK5 kinase may not fully phenocopy genetic perturbation due to its regulation by mechanisms that are independent of autophosphorylation. Furthermore, ERK5 may have scaffolding roles that would be disrupted by gene silencing or deletion but which may not be impacted by inhibition of its kinase activity.^46^

## Evolution of Inhibitors of the ERK5 Kinase Domain

**Figure 2:**
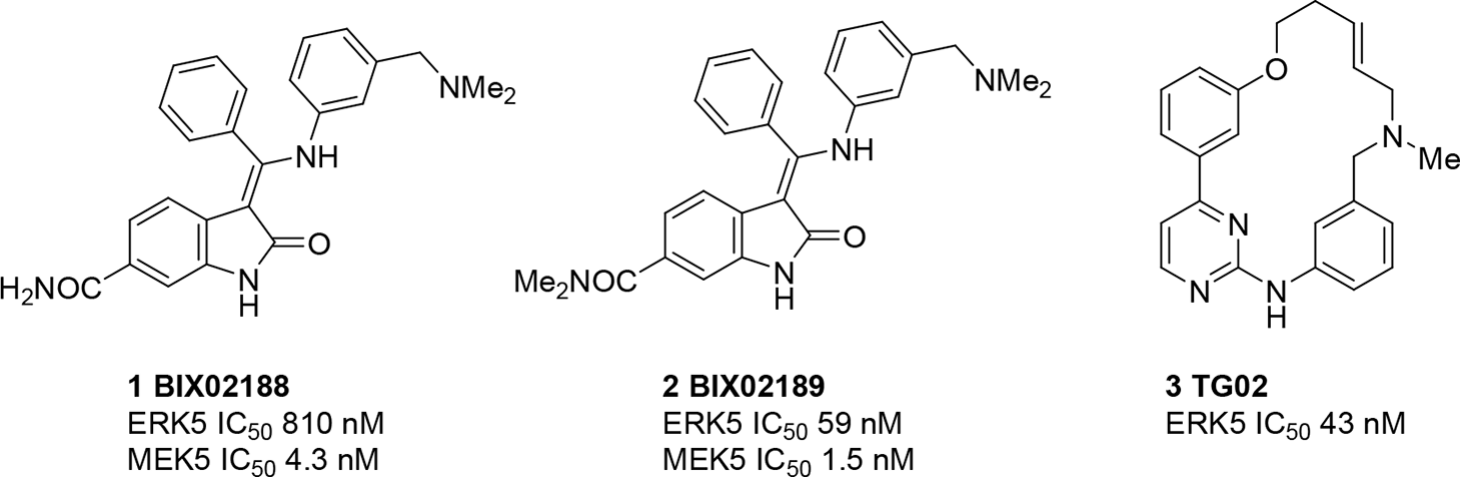
Structures of BIX02188 (**1**), BIX02189 (**2**), and TG02 (**3**).

The first reported inhibitors of the ERK5 kinase domain were indolinone carboxamides BIX02188 (**1**) and BIX01289 (**2**), which were identified from a high-throughput screening (HTS) campaign at Boehringer Ingelheim (Figure 2).^47^ These compounds were selective over MEK1, MEK2, ERK2, and JNK2, exhibited high selectivity over a panel of 87 kinases, but were significantly more potent against MEK5 than ERK5. Neither of these compounds affected phosphorylation of ERK1/2, p38, or Jnk1/2 MAP kinases in HeLa cells but inhibited transcriptional activation of MEF2C, a downstream target of the MEK5/ERK5 pathway, supporting the hypothesis that their effects are due to inhibition of this pathway. These were the first tool compounds to allow interrogation of the MEK5/ERK5 pathways independently of the ERK1/2 pathway.

A macrocyclic inhibitor of ERK5 (TG02, **3**) was reported in 2013 and found to induce a dose-dependent decrease in cell viability on multiple myeloma cell lines.^48^ Although a potent ERK5 inhibitor (*K*_d_ 43 nM), TG02 is however a nonselective inhibitor with low nanomolar inhibitory activity against an array of kinases, including cell cycle regulators CDK1 and CDK2 and transcriptional regulators CDK7 and CDK9. It was therefore not possible to determine the effects of ERK5 inhibition using this compound.

**Figure 3:**
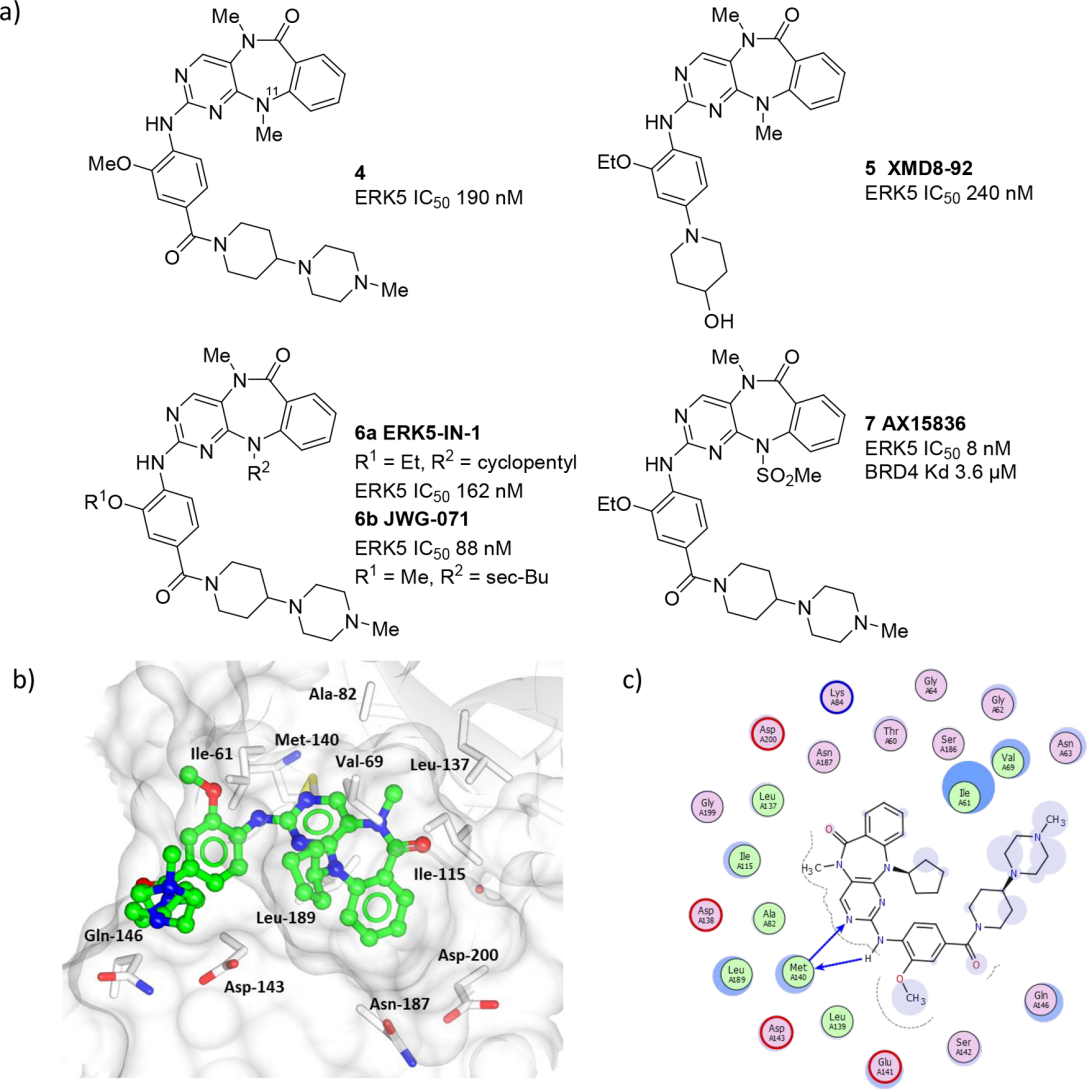
(a) The structure of selected diazepinone ERK5 inhibitors. (b) X-ray cocrystal structure of a diazepinone inhibitor bound to ERK5 (PDB accession code 4B99). (c) Interaction map of **4** with ERK5. Key: (blue arrow) main chain donor/acceptor, (green circle) grease, (pink circle/blue outline/red outline) polar/basic/acidic, (circle blue eclipse) residue protection, (blue circle) solvent exposure.

A significant breakthrough in the field arrived with the discovery of diazepinone inhibitors of the ERK5 kinase domain, which were initially discovered through screening a kinase-targeted compound set in a panel of kinases to furnish **4** (Figure 3).^49^ Further optimization of the arylpiperazine substructure resulted in the identification of XMD8-92 (**5**), a potent ERK5 inhibitor with oral bioavailability in a mouse of 68%.^50^ The binding mode of this series has been elucidated by X-ray crystallography (Figure 3b and 3c).^51^ The aminopyridine of the tricyclic ring system forms a key interaction with the hinge region of the ERK5 ATP binding site. The *N*-11 substituent projects toward the glycine-rich loop, and the carbonyl of the diazepinone central ring forms a hydrogen bond through a water molecule to the backbone nitrogen of D200 in the DFG motif. The piperinylpiperazine ring system extends out of the binding site toward solvent and appears to lie close to the glycine-rich loop.^49^

While XMD8-92 has been widely used as an ERK5 inhibitor tool compound, off-target activity against the BET bromodomain family member BRD4 and leucine-rich repeat kinase 2 (LRRK2) complicates analysis of the results of these studies. Selectivity over LRRK2 was introduced through increasing the size of the substituent on *N*-11 of the diazepinone ring system to give ERK 5-in-1 (**6a**), which resulted in a 30-fold decrease in LRRK2 activity. ERK 5-in-1 has a KINOMEscan selectivity score of 0.007 (3/442), with activity only detected for doublecortin and CaM kinase-like 2 (DCAMKL2) and polo-like kinase 4 (PLK4).^52^ In vivo in mice ERK 5-in-1 has 90% oral bioavailability and a half-life of 8.2 h. However, ERK 5-in-1 has also been shown to have activity against BRD4, with measured IC_50_ values of 200–700 nM.

A recently described ERK5 inhibitor JWG-071 (**6b**) is also based on the diazepinone scaffold with the addition of a larger *sec*-butyl substituent. JWG-071 exhibits greatly (>10-fold) improved selectivity for ERK5 over BRD4 in comparison to XMD8-92 but retains much of XMD8-92’s potency toward other kinases including LRRK2, DCAMKL1, and PLK4.^53,18^ The improved selectivity over BRD4 has been attributed to steric clash of the *sec*-butyl group with the αC-helix of BRD4. Interestingly, JWG-071 has demonstrated tumor-suppressive activity in endometrial cancer xenografts with intraperitoneal administration.^21^

This series was further developed by a different research group, leading to the identification of AX15836 (**7**), through introduction of a methyl sulfonamide on the *N*-11 nitrogen.^54^ This resulted in an ERK5 inhibitor with improved selectivity over BRD4. However, the solubility of AX15836 may compromise its use in some settings, as it has been observed that it exhibits a strong inhibitory effect in the cellular assays with an IC_50_ of 86 nM, but at higher concentrations, activity was lost, possibly due to compound precipitation. AX15836 had no significant effect on cell growth in an IL-6-dependent proliferation assay, surmising that the antiproliferative activity previously reported for AX15836 in a MM.1S cell line that overexpressed a dominant negative ERK5 mutant may be due to non-ERK5-related activity of that compound.^54^

This same study compared the effectiveness of BRD4 inhibitors JQ1 and I-BET762, dual-ERK5-BRD4 inhibitors including XMD8-92 (**5**), and the ERK5-selective inhibitor AX15836 (**7**) in inhibiting proliferation of the acute myeloid leukemia MV-4-11, which contains a mutation reported to lead to constitutive activation of ERK5. BRD4 inhibitors and ERK5-BRD4 dual inhibitors both demonstrated antiproliferative activity in this model, whereas the ERK5-selective inhibitor did not. This data suggests that inhibition of ERK5 kinase activity may not be sufficient or necessary to affect cancer cell proliferation and viability.

**Figure 4:**
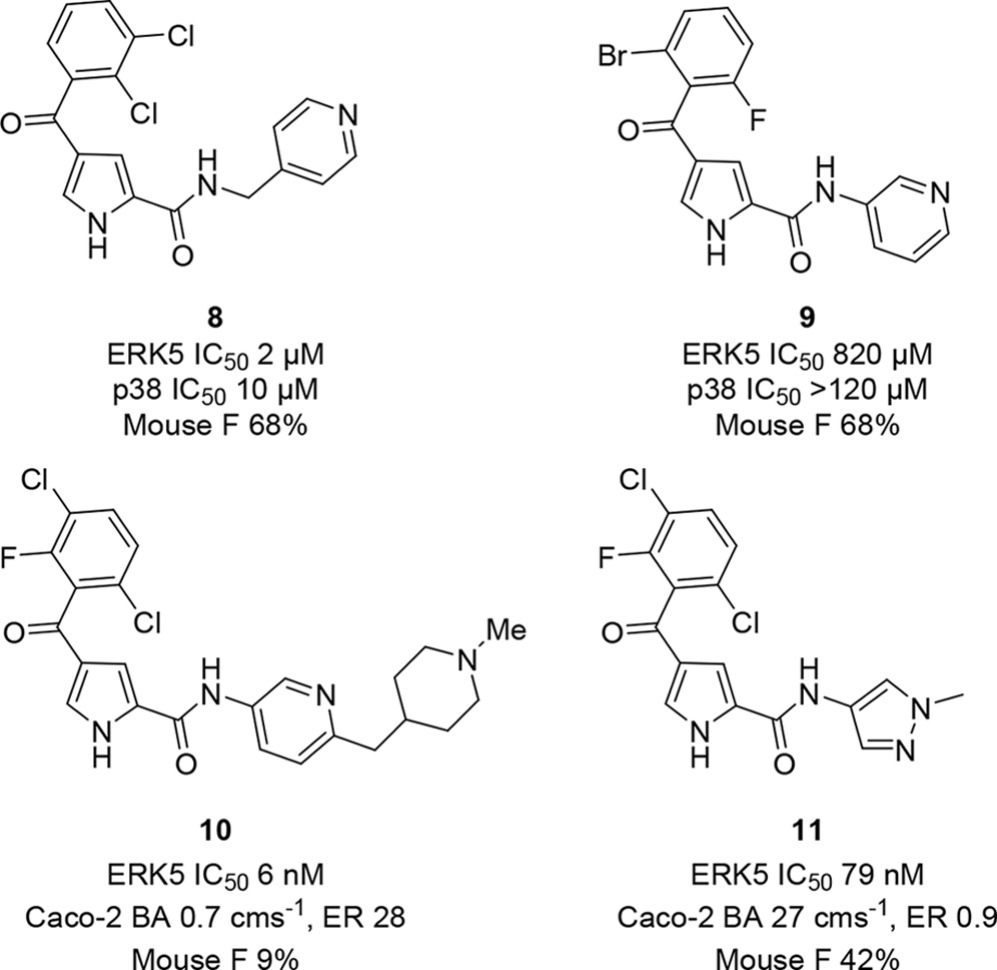
Structures of selected pyrrole carboxamide ERK5 inhibitors.

**Figure 5:**
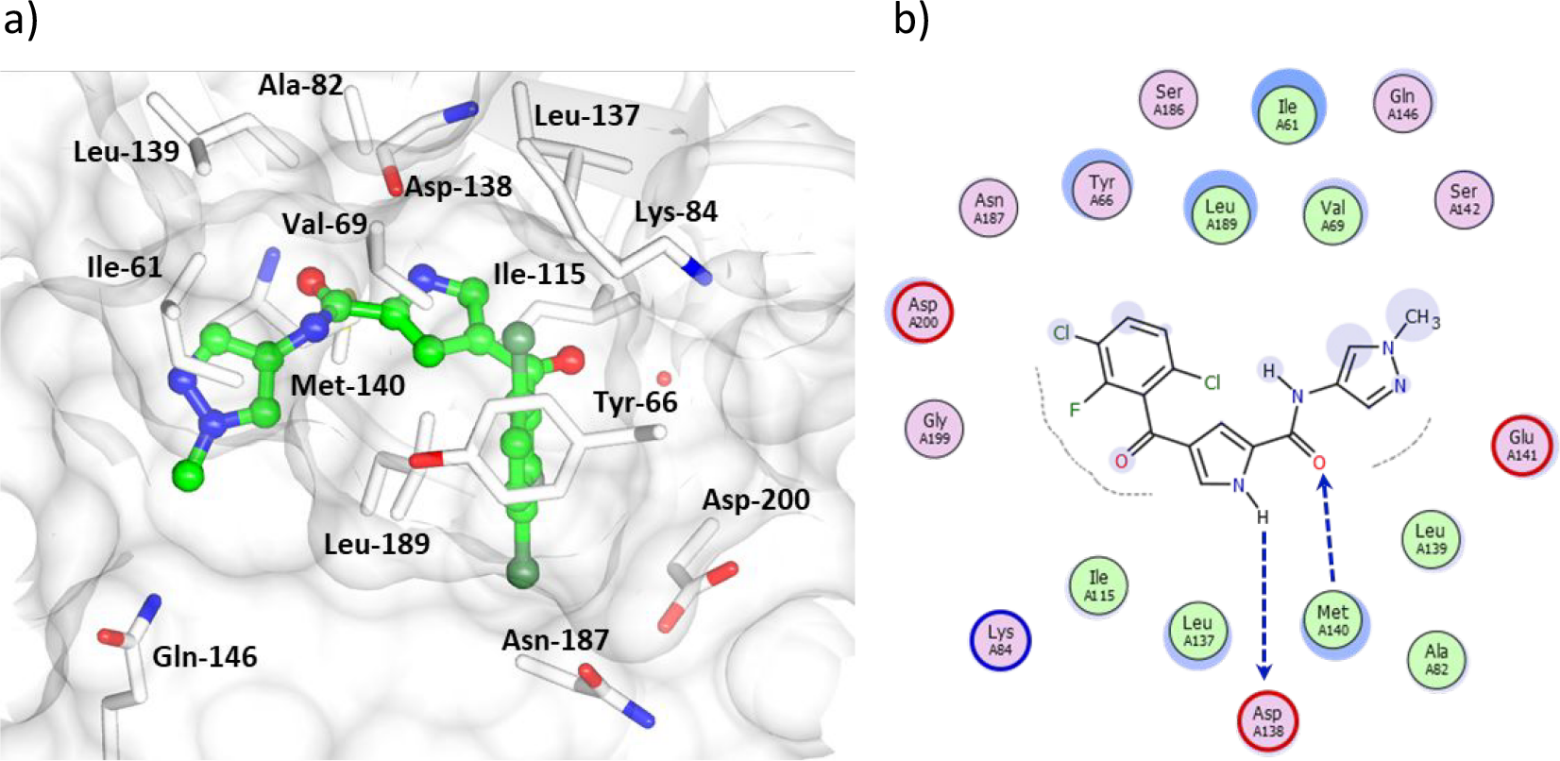
(a) X-ray cocrystal structure of a pyrrole carboxamide inhibitor **11** bound to ERK5 (PDB accession code 7PUS). (b) Interaction map of pyrrole carboxamide inhibitor **11** with ERK5. Key: (blue arrow) main chain donor/acceptor, (green circle) grease, (pink circle/blue outline/red outline) polar/basic/acidic, (circle blue eclipse) residue protection, (blue circle) solvent exposure, (dashes) substitution contour.

A series of pyrrole carboxamide ERK5 kinase domain inhibitors has been disclosed (Figure 4).^55,56^ This class of inhibitors was derived from an HTS campaign. Removal of the methylene spacer in the benzylic amide of HTS hit **8** improved selectivity against the closely related p38 MAP kinase (**9**). Further elaboration of the amide substituent by appending a basic aliphatic heterocycle onto the aryl amide enabled identification of highly potent inhibitors such as **10** (*K*_d_ < 10 nM).^28^ However, the pharmacokinetic parameters were compromised for these basic compounds with low oral bioavailability due to poor passive permeability and high efflux. The *N*-methylpyrazole amide **11** subsequently exhibited the best balance of potency and pharmacokinetics. This compound was also selective over BRD4, although in the DiscoverX KINOMEscan kinase selectivity panel it inhibited several other kinases. *K*_d_ determinations were made for 10 of these kinases (CSF1R *K*_d_ 46 nM, DCLK1 *K*_d_ 61 nM, MAPK7 *K*_d_ 180 nM, LRRK2 *K*_d_ 220 nM, AURKA *K*_d_ 290 nM, FGFR1 *K*_d_ 380 nM, KIT *K*_d_ 420 nM, ABL1 *K*_d_ 1.2 μM, JAK3 *K*_d_ 1.3 μM, and MEK5 *K*_d_ 2.8 μM). Activity against these kinases should be taken into consideration when interpreting results from biological studies using this compound, as off-target activities could influence observed results (e.g., inflammation or angiogenesis). The binding mode of this series was determined by X-ray crystallography of **11** in complex with ERK5 at a resolution of 2.75 Å (Figure 5). The pyrrole carboxamide forms a bidentate interaction with the hinge region with the methylpyrazole amide lying between the side chains of E146, M140, and I61 in a small channel at a solvent-exposed region of the binding pocket. The halogenated ring occupies a buried hydrophobic pocket.

**Figure 6:**
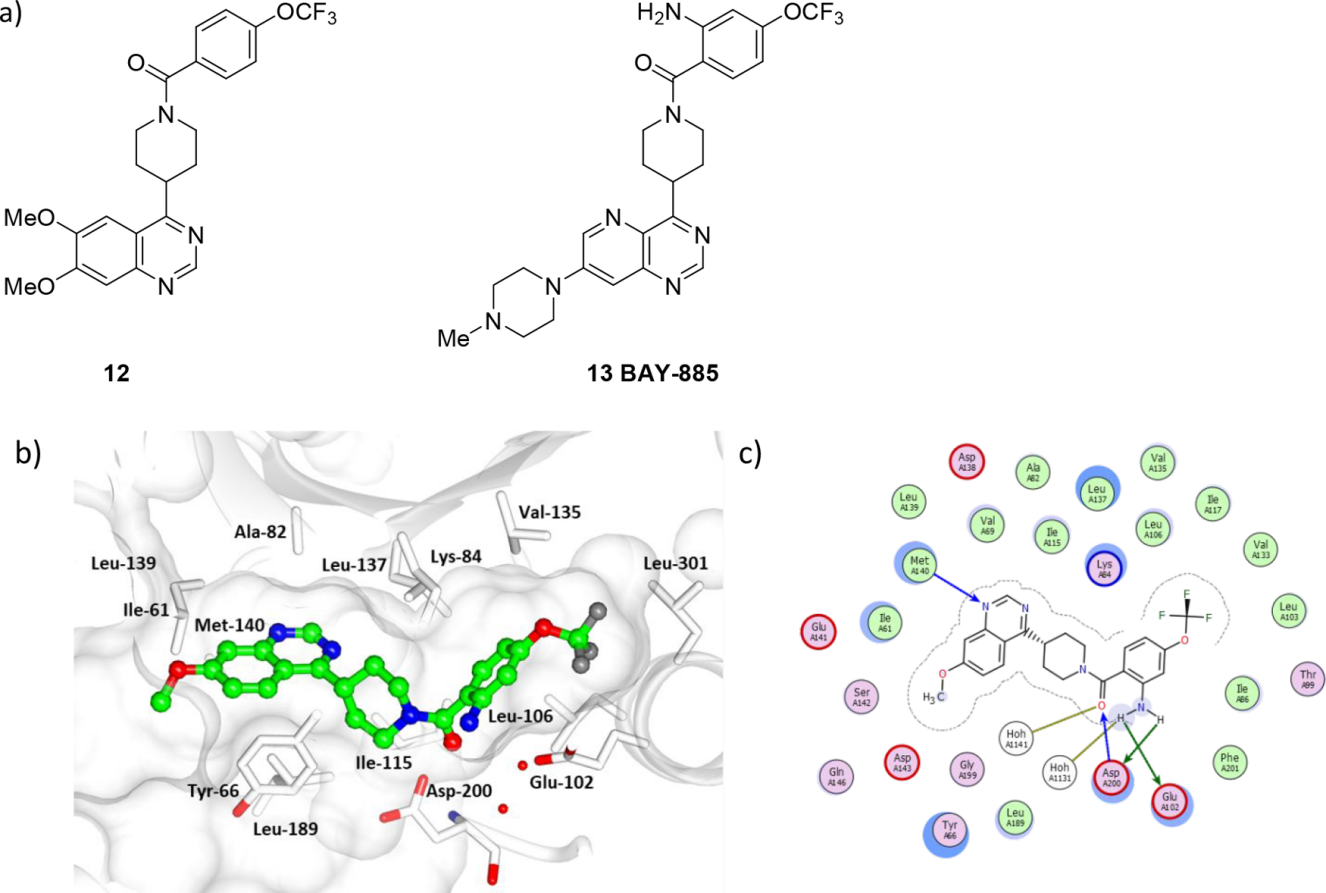
(a) The structures of quinazoline-based ERK5 inhibitors **12** and **13** (BAY-885). (b) X-ray cocrystal structure of a quinazoline scaffold bound to ERK5 (PDB accession code 6HKN). (c) Interaction map of quinazoline inhibitor with ERK5. Key: (blue arrow) main chain donor/acceptor, (green arrow) side chain donor/acceptor, (gold arrow) water H bond, (white circle) water, (green circle) grease, (pink circle/blue outline/red outline) polar/basic/acidic, (circle blue eclipse) residue protection, (blue circle) solvent exposure, (dashes) substitution contour.

**Figure 7:**
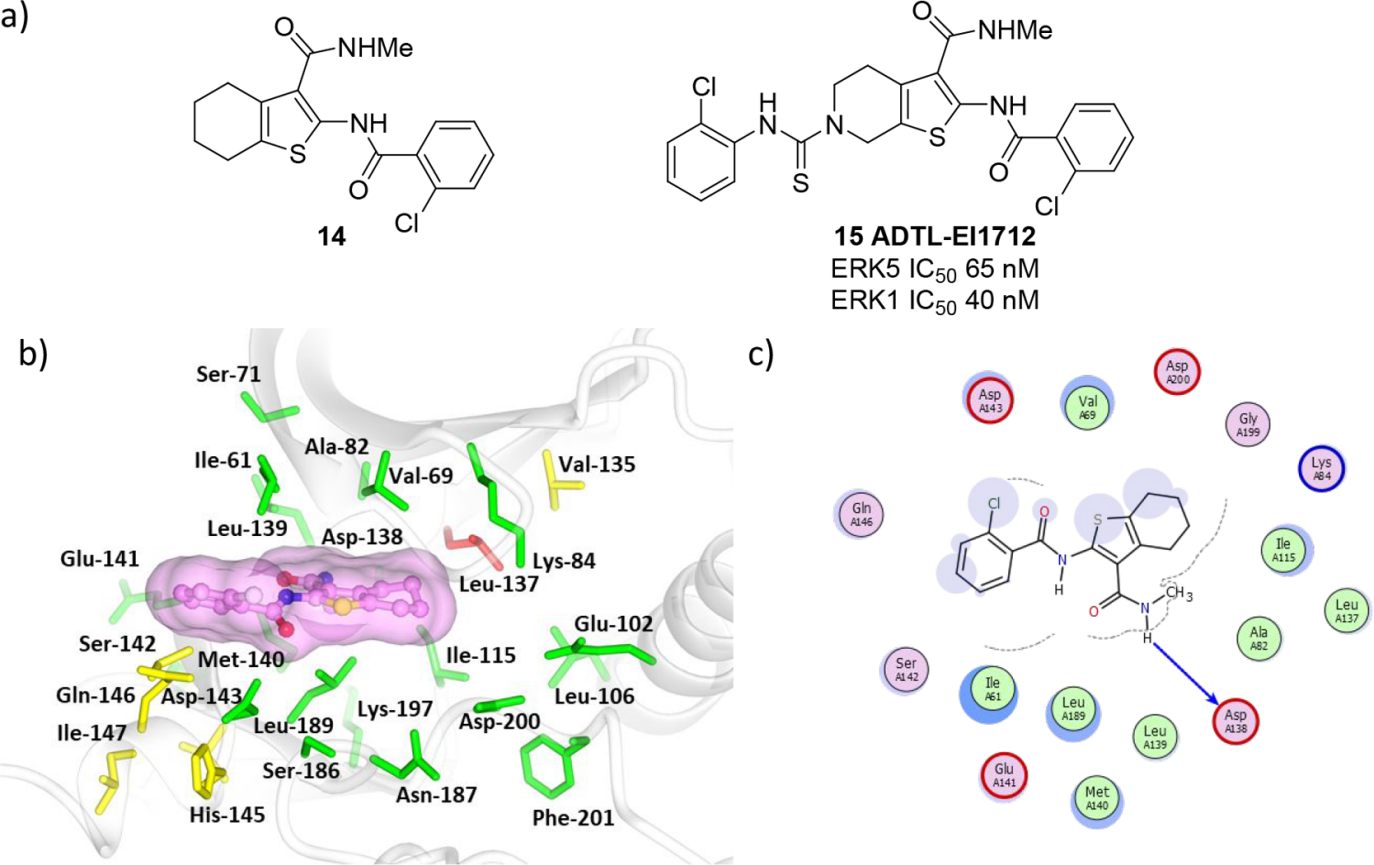
(a) The structures of thiophene dual-target inhibitors **14** and **15** (ADTL-EI1712). (b) Model of **14** bound to the active site of ERK5 using PDB accession code 4B99. Residues within 8 Å of **14** are colored using sequence conservation against ERK1 (green, conserved; red, nonconserved). (c) Potential interaction map of compound **14** bound with ERK5. Key: (blue arrow) main chain donor/acceptor, (green circle) grease, (pink circle/blue outline/red outline) polar/basic/acidic, (circle blue eclipse) residue protection, (blue circle) solvent exposure, (dashes) substitution contour.

A quinazoline-based series of ERK5 inhibitors has been developed from high-throughput screening hit **12** through structure-guided optimization into BAY-885 (**13**), a selective ERK5 kinase domain inhibitor with high solubility and membrane permeability (Figure 6a).^57^ In the X-ray crystal structure of BAY-885 (**13**) bound to ERK5 (Figure 6b and 6c), the quinazoline *N*-1 nitrogen forms a hydrogen bond with the backbone NH of L139 in the hinge region (Figure 7). The amide carbonyl forms a hydrogen bond with D200, and the benzamide aryl occupies a hydrophobic pocket. The amino group forms a hydrogen-bond network with K84, D200, and Q102.

BAY-885 (**13**) was shown to be chemically stable across the pH range with no *h*ERG inhibition at 10 μM and low to moderate in vitro metabolic stability in rat hepatocyes and human liver microsomes.^57^ BAY-885 demonstrated potent kinase and transcriptional activation inhibition in the SN12C-MEF2 reporter cell line (IC_50_ = 115 nM/IC_90_ = 691 nM). In a panel of 358 kinases (Eurofins kinase panel) at 1 μM compound concentration only 3 kinases (*r*Fer, *h*EPhB3, and *h*Eph5A) were inhibited at >20% and exhibited no binding to BRD4 at 20 μM. BAY-885 failed to inhibit cellular proliferation in cells with ERK5 genomic amplification (SN12C, SNU-449, MFM-223) or with constitutively active ERK5 signaling (BT-474, SK-BR-3).^57^ These results were proposed to support that ERK5 kinase activity is dispensable for cancer cell growth and questioning the therapeutic potential of ERK5 kinase inhibitors for anticancer drug development.

In developing inhibitors of the ERK5 kinase domain, selectivity over kinases and nonkinase targets such as BRD4 initially confounded the understanding of ERK5 pharmacology. However, selectivity hurdles have been overcome, and multiple orthogonal chemotypes of inhibitors of the ERK5 kinase domain have now been identified. Obtaining pharmacokinetic profiles suitable for in vivo dosing in animal models has also been achieved, but conflicting reports on efficacy in such models has decreased confidence in the use of ERK5 inhibitors as single-agent chemotherapeutics. Attention next pivoted toward ERK5 inhibitors as combination therapies or single agents with defined multiple pharmacological inhibition. Recently published patents disclose results from biological studies of previously unpublished ERK5 inhibitors in combination with a range of existing compounds against other cancer-related target proteins including inhibitors of MEK1/2, TEAD, and existing chemotherapeutics including sorafenib, osimertinib, palbociclib, trametinib, and MRTX1133.^58^ This may suggest that while inhibition of ERK5 kinase activity in isolation may not be sufficient to affect cancer pharmacology, more significant effects may be achievable through the use of ERK5 inhibitors as part of a combination regimen.

## Dual-Pharmacology Inhibitors

Resistance to agents that modulate the RAF-MEK1/2-ERK1/2 may develop due to compensatory activation of other pro-survival/proliferative signals, including receptor tyrosine kinases and PI3K. Activation of the MEK5–ERK5 pathway has also been proposed as a resistance mechanism to RAF-MEK1/2-ERK1/2 inhibitors.^59^ Several proteins, such as c-Myc and RSK, are substrates for phosphorylation by both ERK1 and ERK5, leading to induction of c-Jun or c-Fos genes.^27,60^ Identification of compounds which inhibit both ERK1/2 and ERK5 has therefore been investigated.^61^ A rapid docking protocol was first used for virtual screening of a 97 000 compound library in ERK1 (PDB 4QTB) which identified 1000 hit compounds. The hit compounds were subsequently docked using a more accurate docking algorithm into both ERK1 and ERK5 (PDB 4B99). The 15 resulting dual ERK1/ERK5 hits were screened in a biochemical assay, leading to the identification of compound **14** as a validated dual-pharmacology hit (Figure 7).

Replacement of the cyclohexane with a piperidine opened a vector for growing the hit into a hydrophobic pocket. This resulted in the identification of **15** (ADTL-EI1712), which inhibited ERK1 (IC_50_ 40 nM) and ERK5 (IC_50_ 65 nM). Tumor growth was inhibited by ADTL-EI1712 in a mouse xenograft model, but massive cytoplasmic vacuolization was induced in MKN-74 cells, which suggests that the compound may have other off-target pharmacology. However, ADTL-EI1712 provides proof of concept that dual inhibition of ERK1/2 and ERK5 can be achieved in a single molecule, and further work in this area may prove fruitful.

Crosstalk between the PI3K/protein kinase B (Akt) and the MEK1/2-ERK1/2 signaling pathways may play a role in cancer, leading to therapeutic strategies involving combinations of inhibitors to target both. However, efficacy and safety profiles of these approaches have to date been compromised. The MEK5/ERK5 pathway has also been linked to Akt signaling, with Akt being found to phosphorylate MEKK3, the upstream kinase of MEK5.^62^ Combinations of the pan-Akt ATP-site inhibitor ipatasertib and the ERK5 inhibitors XMD8-92 and AX15836 have been investigated in triple-negative breast cancer cell lines.^63^ In this study, ipatasertib in combination with XMD8-92 synergistically decreased TNBC cell viability while sparing MCF-10 cells. The possibility of the off-target BRD4 inhibitory activity of XMD8-92 was controlled for by also examining combinations with the more selective ERK5 inhibitor AX15836 for their effect on c-Myc expression. AX15836 was unable to reduce c-Myc expression significantly in MDA-MB-231 cells. Interestingly, a control selective BRD4 inhibitor (CPI-203) was also unable to reduce c-Myc expression significantly, but a combination of AX15836 and CPI-203 was able to recapitulate the results of XMD8-92, suggesting the activity of the compound may result from inhibiting ERK5 and BRD4 simultaneously. The synergistic activity of the ERK5–Akt inhibitor combination may be attributable to a dual effect on the pro-apoptotic protein Bad. ERK5 and Akt both regulate phosphorylation of Bad (at Ser112 and Ser136, respectively), the inhibition of which would confer a susceptibility to apoptosis.^63^

## Beyond Inhibition of the Kinase Domain

Recently, the complexities of ERK5 inhibitor pharmacology have been brought into sharper focus by the discovery that ERK5 transcriptional activity can increase upon binding of kinase inhibitors. ERK5 kinase inhibitor binding has been shown to cause a conformational change that dissociates the NLS and TAD, leading to translocation of ERK5 to the nucleus and stimulation of KLF2, an ERK5 regulated promoter.^64^ Thus, the binding of an ERK5 inhibitor can lead to unanticipated activation of downstream gene transcription, a phenomenon that has been named paradoxical activation.^18,64^ ERK5 inhibitors from different structural classes have, to differing degrees, been shown to induce paradoxical activation, including the selective diazepinone ERK5 inhibitor **7** (AX15836), pyrrole carboxamide **11**, aza-quinazoline **13** (BAY-885), and thiophene **15** (ADTL-EI1712). These paradoxical activators all form classical hydrogen-bonding interactions to the hinge region of the ATP binding site. BAY-885 and its analogues also occupy the back pocket of the kinase, located between the αC helix and the L137 gatekeeper residue. This unexpected transcriptional activation could severely limit the therapeutic applications of ERK5 kinase inhibitors and suggests that alternative strategies are required to maximally inhibit ERK5 activity.

**Figure 8:**
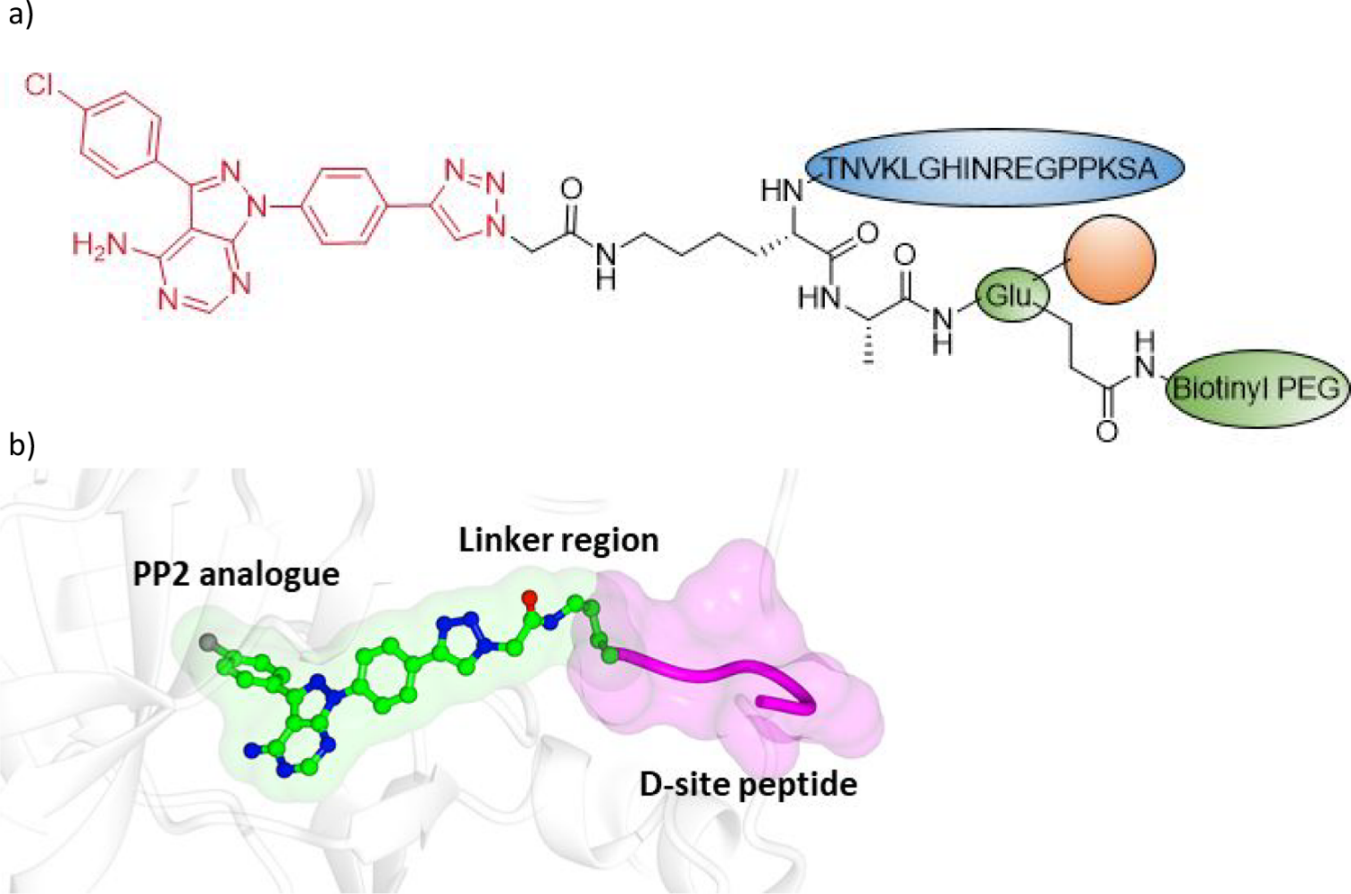
Bivalent Inhibitor design. (a) Structure of **16** (ERK5.1). PP2-derived kinase ligand substructure shown in red. The D-site peptide motif is highlighted in blue. (b) Proposed binding mode of **16** bound to ERK5 engaging with the hinge and the D site.

One alternative approach that has been investigated is the development of bivalent molecules that inhibit ERK5 kinase activity and autophosphorylation in a single high molecular weight molecule.^65^ Thus, an aminopyrazolopyrimidine kinase hinge binding motif derived from PP2,^66^ selected due to precedented success in preparation of bifunctional peptide hybrids, was conjugated to a 19-amino acid residue D-site peptide motif derived from the sequence of MEK5 to attempt to block the MEK5–ERK5 interaction. Compound design was facilitated by analysis of the ERK5–MEK5 crystal structure (PDB: 4IC7) and prospective docking of linked compounds to identify optimal linking vectors and lengths. The Huisgen Click chemistry used to couple the bivalent molecules together allowed several linker lengths to be explored, with the shortest linker investigated (ERK5.1, **16**, Figure 8) being the only compound to demonstrate superior binding when compared to the control monomer structures. Longer linkers resulted in compounds with similar binding affinity to the monomers, indicating that correct linker length was critical to finding compounds with cooperative binding.

ERK5.1 was shown to inhibit ERK5 phosphorylation in cell-based assays, whereas the monomers and bivalent compounds with longer linkers did not, suggesting that only ERK5.1 is able to block the MEK5–ERK5 interaction by blocking the D-site binding groove. This also confirmed that these large molecular weight peptide analogues were cell penetrant. Interestingly, in a kinase inhibition assay, PP2 alone was unable to antagonize ERK5 autophosphorylation, whereas ERK5.1 was a potent antagonist that was also selective over ERK1 and ERK2.^65^

ERK5.1 significantly inhibited cancer stem cell colony formation at 5 and 10 μM, and in a wound/scratch assay, ERK5.1 significantly inhibited cell migration and wound healing at 10 μM, whereas the monomer controls did not. This study demonstrates that alternative pharmacological outcomes can be achieved by inhibiting both ERK5 kinase activity and the MEK5-ERK5 protein–protein interaction in a single molecule.

**Figure 9:**
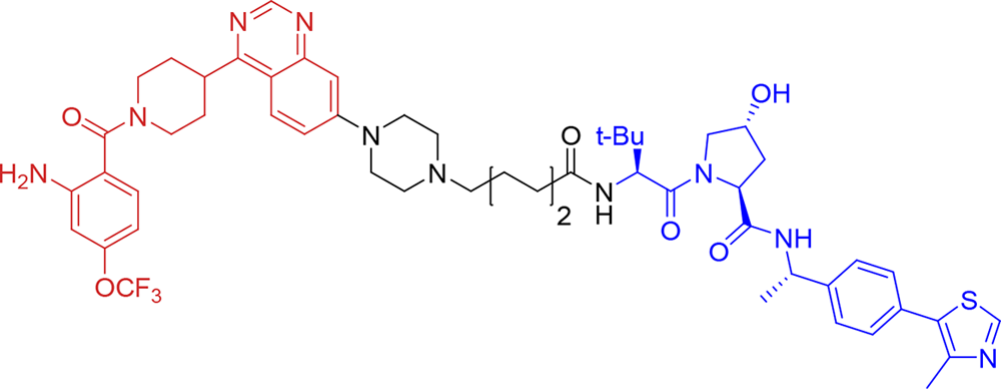
Structure of ERK5 bifunctional degrader **17** (INY-06-061). Quinazoline-based ERK kinase inhibitor motif shown in red, linker shown in black, and VHL ligand shown in blue.

Proteolysis-targeting chimeras (PROTACs) have emerged as powerful tools for inducing degradation of a protein through proximity-induced ubiquitination of the protein by a ubiquitin E3 ligase enzyme in order to effect a biological response.^67^ The development of PROTAC degraders of ERK5 has recently been disclosed^68^ and has provided some interesting results that provide further insight into the biological consequences of modulation of ERK5 protein levels. An ERK5 bifunctional degrader should reduce both the kinase activity and the nonenzymatic functions (including transcriptional activation) of ERK5 within a single molecule and would therefore be expected to recapitulate the antiproliferative effects of ERK5 gene knockdown more effectively. Structure-based design of PROTAC molecules enabled the identification of INY-06-061 (**17**, Figure 9) through linking a quinazoline-based ERK5 kinase domain inhibitor to a von Hippel Lindau (VHL) E3 ligase ligand using a piperazine alkylamide linker. INY-06-061 was confirmed as a dose-dependent and proteasome-dependent degrader of ERK5 (DC_50_ 21 nM). INY-06-061 had no degrading effect on 7700 other proteins quantified in the MOLT2 cell line used, suggesting it is a highly selective ERK5 degrader.

In a panel of 750 cell lines, acute degradation of ERK5 with INY-06-061 did not result in decreased proliferation (all IC_50_ values exceeded 1 μM), and in IL-6-stimulated multiple myeloma MM.1S cells, INY-06-061 also had no antiproliferative effect. In primary endothelial cells, INY-06-061 had no effect on the inflammatory cytokine response following stimulation with lipopolysaccharide, despite this being reported with siRNA to ERK5.^54^ Further studies are warranted to explore the effect of degree and duration of target manipulation with bifunctional degraders, particularly with molecules that are suitable for use in vivo. However, the published studies exploring chemically induced degradation of ERK5 protein cast further doubt on ERK5 having a major role in driving cancer cell proliferation, in contrast to studies that examined gene silencing of *MAPK7* with siRNA.

Targeting ERK5 splicing at the RNA level may provide another angle to modulate ERK5 transcriptional activity. Splice variants of ERK5 have been identified, which affect subcellular localization and consequently influence pharmacological activity. Changes in the 5′UTR introduce a premature stop codon leading to shorter *N*-terminal truncated ERK5 variants in mice (mERK5b and mERK5c). This results in exclusive nuclear localization through removal of the cytoplasmic localization sequence. These variants have no kinase activity and are unable to bind ATP or to associate with MEK5^7^ and inhibit ERK5 kinase activity and ERK5-mediated MEF2C transactivation. Another splice variant ERK5-T (ERK5-Truncated) results from the retention of intron 4. This variant lacks the NLS and the PR domains. This variant is phosphorylated by MEK5 but does not translocate to the nucleus upon phosphorylation and also impairs the translocation of full-length ERK5.^69^ Thus, targeting alternative splice variants of ERK5 with RNA-targeted ligands may provide another approach to modulate ERK5 signaling.

## Conclusion

Research into modulation of ERK5 and its potential as an anticancer therapeutic strategy continues to evolve. The initial rationale of inhibiting ERK5 kinase activity has faced barriers of kinase selectivity, selectivity over nonkinases such as BRD4, and development of inhibitors with suitable pharmacokinetics for in vivo dosing. Preclinical studies of early inhibitors yielded ambiguous results, which in part was attributed to poor selectivity over other kinases and nonkinase off targets. However, several chemotypes with orthogonal selectivity profiles have now been identified. Results in disease models continue to give conflicting results, much of which may now be attributed to the paradoxical activation of the *C*-terminal transcriptional activity by ERK5 kinase inhibitors. The data provide increasing evidence that deriving therapeutic benefit from the modulation of ERK5 by small molecules is likely to require more sophisticated approaches than single-agent orthosteric kinase domain inhibition. Recent publication of the biological effects of a bifunctional degrader of ERK5 which indicated that this failed to induce a strong antiproliferative effect in cancer cell lines is in contrast to a number of studies examining genetic perturbation of ERK5 with some of the discrepancy potentially being a consequence of utilizing siRNA with a lack of selectivity or incomplete degradation of ERK5 using PROTACs.^68^ Further studies on the effect of chemical-induced ERK5 degradation are warranted, particularly in vivo (e.g., the effect on angiogenesis or cancer cell metastasis), to further elucidate the biological functions of ERK5. Additional work to investigate alternative approaches to ERK5 inhibition, such as modulating ERK5 splicing, may also be useful in the search for productive therapeutic strategies.

## Supporting Information Available

Molecular formula strings (CSV)

## Abbreviations Used

BET: bromodomain and extraterminal motif
BMK: big MAP kinase
BRD: bromodomain-containing protein
CA: calmodulin-dependent protein kinase
FAK: focal adhesion kinase
JNK: Janus kinase
lklf: lung kruppel-like factor
LRRK: leucine-rich repeat kinase
MEF: myocyte-enhancer factor
MEK: mitogen-activated protein kinase kinase
PLK: polo-like kinase
PR: proline-rich domain
PROTAC: proteolysis-targeting chimera
RSK: ribosomal s6 kinase
TAD: transcriptional activation domain
TNBC: triple-negative breast cancer
UTR: untranslated region
